# The ecology and evolution of wildlife cancers: Applications for management and conservation

**DOI:** 10.1111/eva.12948

**Published:** 2020-03-23

**Authors:** Rodrigo Hamede, Rachel Owen, Hannah Siddle, Sarah Peck, Menna Jones, Antoine M. Dujon, Mathieu Giraudeau, Benjamin Roche, Beata Ujvari, Frédéric Thomas

**Affiliations:** ^1^ School of Natural Sciences University of Tasmania Hobart Tas. Australia; ^2^ Centre for Integrative Ecology School of Life and Environmental Sciences Deakin University Vic. Australia; ^3^ Centre for Biological Sciences University of Southampton Southampton UK; ^4^ Wildlife Veterinarian, Veterinary Register of Tasmania South Hobart Tas. Australia; ^5^ Centre de Recherches Ecologiques et Evolutives sur le Cancer/Centre de Recherches en Ecologie et Evolution de la Santé Unité Mixte de Recherches Institut de Recherches pour le Développement 224‐Centre National de la Recherche Scientifique 5290‐Université de Montpellier Montpellier France

**Keywords:** cancer, disease ecology, host–pathogen interactions, natural selection, transmissible tumour, wildlife management

## Abstract

Ecological and evolutionary concepts have been widely adopted to understand host–pathogen dynamics, and more recently, integrated into wildlife disease management. Cancer is a ubiquitous disease that affects most metazoan species; however, the role of oncogenic phenomena in eco‐evolutionary processes and its implications for wildlife management and conservation remains undeveloped. Despite the pervasive nature of cancer across taxa, our ability to detect its occurrence, progression and prevalence in wildlife populations is constrained due to logistic and diagnostic limitations, which suggests that most cancers in the wild are unreported and understudied. Nevertheless, an increasing number of virus‐associated and directly transmissible cancers in terrestrial and aquatic environments have been detected. Furthermore, anthropogenic activities and sudden environmental changes are increasingly associated with cancer incidence in wildlife. This highlights the need to upscale surveillance efforts, collection of critical data and developing novel approaches for studying the emergence and evolution of cancers in the wild. Here, we discuss the relevance of malignant cells as important agents of selection and offer a holistic framework to understand the interplay of ecological, epidemiological and evolutionary dynamics of cancer in wildlife. We use a directly transmissible cancer (devil facial tumour disease) as a model system to reveal the potential evolutionary dynamics and broader ecological effects of cancer epidemics in wildlife. We provide further examples of tumour–host interactions and trade‐offs that may lead to changes in life histories, and epidemiological and population dynamics. Within this framework, we explore immunological strategies at the individual level as well as transgenerational adaptations at the population level. Then, we highlight the need to integrate multiple disciplines to undertake comparative cancer research at the human–domestic–wildlife interface and their environments. Finally, we suggest strategies for screening cancer incidence in wildlife and discuss how to integrate ecological and evolutionary concepts in the management of current and future cancer epizootics.

## 1 INTRODUCTION

Over the last two decades, significant efforts have been made to incorporate ecological and evolutionary principles to better understand the dynamics of wildlife diseases and their impact on wild populations (Galvani, 2003; Tompkins, Dunn, Smith, & Telfer, 2011; Vander Wal et al., 2014). The reciprocal interactions between host and pathogens are in many ways analogous to the interplay of ecological and evolutionary processes between species and their environment. Thus, the eco‐evolutionary processes and feedbacks in emerging host–pathogen systems are currently considered key in epidemiology and disease management (Brosi, Delaplane, Boots, & Roode, 2017; Coen & Bishop, 2015; Grenfell et al., 2004). Cancer is a disease that evolved with the transition to multicellularity (Aktipis & Nesse, 2013) and therefore affects most metazoans on earth. It corresponds to a family of potentially lethal pathologies in which normal cells lose their typical cooperative behaviour, proliferate, spread and hence become malignant. Despite the ubiquitous nature of cancers in wildlife, the role of the oncobiota (i.e. oncogenic phenomena from precancerous lesions to metastatic cancer, Thomas et al., 2017) in ecological and evolutionary processes has been historically neglected (but see Thomas et al., 2017; Vittecoq et al., 2013) and its applications for wildlife management and conservation remain mostly in their infancy. Given that cancer is an evolving disease where the ecological context of tumour–host interactions is of paramount relevance for disease progression and immunological responses, evolutionary principles have recently been used in oncology as a novel approach for developing therapeutic treatments (Enriquez‐Navas, Wojtkowiak, & Gatenby, 2015; Willyard, 2016; Zhang, Cunningham, Brown, & Gatenby, 2017).

Oncogenic phenomena can act as important agents of selection by having differential effects on the survival, life history, reproductive success and fitness of hosts (Thomas et al., 2017, 2018; Ujvari, Beckmann, et al., 2016). These processes can shape phenotypic, genetic and epigenetic variance across individuals, populations and species. Carcinogenesis is a complex process that depends on trade‐offs at the cellular and organismal levels, and, in turn, these trade‐offs interact with individuals and species, and hence ecosystems (Jacqueline et al., 2017; Pesavento, Agnew, Keel, & Woolard, 2018; Wu, Wang, Ling, & Lu, 2016). Thus, cancer should not be studied in isolation but as an interacting force of selection between species and their changing environments. Furthermore, in a century characterized by rapid environmental changes, species are increasingly facing additional ecological and immunological trade‐offs that in turn may increase cancer risk (Jacqueline et al., 2017). Unravelling the synergistic effects of environmental degradation, ecological and evolutionary processes, and susceptibility to cancer is nonetheless a complex task. Recognizing these complexities using a multidisciplinary approach will permit the understanding of important concepts underpinning cancer emergence and evolution and at the same time identify novel and integrative frameworks for managing cancers in wildlife.

The misleading assumption that cancers in wildlife are rare stems from the logistic difficulties in detecting their occurrence and monitoring their prevalence: in most cases, afflicted hosts are preyed upon or die unseen (Vittecoq et al., 2013). This suggests that most cancers in the wild are unreported and understudied. In addition, infectious agents are now well recognized as important drivers of cancer causation. For example, 15%–20% of all cancers in humans have been associated with a direct infectious origin (i.e. oncoviruses) (Alizon, Bravo, Farrell, & Roberts, 2019; Ewald & Swain Ewald, 2015). There is considerable evidence that environmental factors are a major contributor to cancer risk. Anthropogenic activities such as urbanization, chemical contamination and knock‐on effects from rapid environmental changes have been associated with high cancer prevalence in wildlife and a lack of upregulation of anticancer defence mechanisms in these carcinogenic habitats (Giraudeau, Sepp, Ujvari, Ewald, & Thomas, 2018; Giraudeau et al., 2020; Pesavento et al., 2018; Sepp, Ujvari, Ewald, Thomas, & Giraudeau, 2019). However, only recently has cancer been considered a disease of conservation concern (McAloose & Newton, 2009) and transmissible cancers regarded as a new modality of infectious disease (Metzger & Goff, 2016). The increasing number of virus‐associated and directly transmitted cancers detected in wildlife (Table 1), particularly for species already endangered (Gulland, Trupkiewicz, Spraker, & Lowenstine, 1996; James et al., 2019; McCallum et al., 2009; Williams et al., 1994; Woolford et al., 2008), demonstrates the urgent need for developing a holistic framework for studying oncogenic phenomena in the wild. Studying patterns of emergence, tumour–host interactions and evolutionary processes between hosts and malignant cells will also provide new insights into our understanding of how cancer defence mechanisms arise and evolve in nature (Nunney, 2013).

**TABLE 1:** Examples of infectious cancer in wildlife populations, their aetiology and prevalence

Taxa	Species	IUCN status	Cancer type	Aetiology	Prevalence	Reference
Amphibian	Leopard frog (*Rana pipens*)	NL	Renal adenocarcinoma	Viral	10%	Granoff (1973)
	Tiger salamander (*Ambystoma tigrinum*)	LC	Pollution‐related melanophoromas	Various pollutants	30%–50%	Rose and Harshbarger (1977)
	Japanese newt (*Cynops pyrrhogaster*)	LC	Cutaenous papillomas	Virus‐like agent	Up to 5.45%	Asashima, Komazaki, Satou, and Oinuma (1982)
	Alpine newt (*Ichthyosaura alpestris*)	LC	Cutaenous papillomas	Unknown	20%–30%	Greven and Guex (2018)
	Montseny brook newt (*Calotriton arnoldi*)	CE	Skin tumour	Unknown	Up to 29%	Martínez‐Silvestre, Amat, Bargalló, and Carranza (2011)
Avian	Barn swallow (*Hirundo rustica*)	LC	Cutaneous masses	Radiation	1.50%	Møller, Bonisoli‐Alquati, and Mousseau (2013)
	White fronted goose (*Anser albifrons*)	LC	Lipoma/fibroma	Unknown	23%	Daoust, Wobeser, Rainnie, and Leighton (1991)
Fish	Walleye *(Sander vitreus*)	LC	Walleye dermal sarcoma	Viral	Unknown	Coffee, Casey, and Bowser (2013)
	Walleye *(Sander vitreus*)	LC	Discrete epidermal hyperplasia	Viral	Unknown	Coffee et al. (2013)
	Chinook salmon (*Oncorhynchus tshawytscha*)	NL	Plasmacytoid leukaemia	Viral	5%	Coffee et al. (2013)
	European smelt (*Osmerus eperlanus*)	LC	Spawning papillomatosis	Viral	5.50%	Coffee et al. (2013)
	Damsel fish (*Stegastes partitus*)	LC	Neurofibromatosis	Virus‐like agent	Up to 23%	Coffee et al. (2013)
	Brown bullhead (*Ameiurus nebulosus*)	LC	Cutaneous papillomas and squamous cell carcinoma	Pollutants	19%–58%	Baumann et al. (2008)
	English sole (*Parophrys vetulus*)	NL	Liver cancer	Pollutants	24%	Malins et al. (1988)
	Northern pike (*Esox lucius*)	LC	Lymphoma	Viral	21%	Papas, Dahlberg, and Sonstegard (1976)
	White sucker (*Catostomus commersonii*)	LC	Squamous cell carcinoma	Pollutants	33%–58%	Blazer et al. (2017)
Mammal	Dusky dolphins (*Lagenorhynchus obscurus*)	LC	Genital papilloma	Viral	57%–74%	Van Bressem, Waerebeek, Piérard, and Desaintes (1996)
	Long beaked dolphin *(Delphinus capensis*)	DD	Genital papilloma	Viral	50%	Van Bressem et al. (1996)
	Bottlenose dolphin (*Tursiops truncatus*)	LC	Genital papilloma	Viral	33%	Van Bressem et al. (1996)
	Burmeister's porpoise (*Phocoena spinipinnis*)	NT	Genital papilloma	Viral	48.50%	Van Bressem et al. (1996)
	Tasmanian devil (*Sarcophilus harrisii*)	E	Transmissible tumour	Transmissible cancer	40%–80%	Pearse and Swift (2006)
	Tasmanian devil (*Sarcophilus harrisii*)	E	Transmissible tumour	Transmissible cancer	20%–40%	Pye, Pemberton, et al. (2016))
	Beluga whale (*Delphinapterus leucas*)	LC	Intestinal adenocarcinoma	Pollutant	27%	Martineau et al. (2002)
	Sperm whale (*Physeter macrocephalus*)	V	Genital papilloma	Viral	10%	Lambertsen, Kohn, Sundberg, and Buergelt (1987)
	Island fox (*Urocyon littoralis catalinae*)	NT	Ceruminous gland hyperplasia/carcinoma	Ectoparasite	50%	Vickers et al. (2015)
	Raccoon (*Procyon lotor*)	LC	Brain tumours	Viral	15%	Cruz et al. (2013)
	Western bandicoot (*Perameles bougainville*)	V	Cutaneous hyperplasia/carcinoma	Viral	9%–18%	Woolford et al. (2008)
	California sea lion (*Zalophus californianus*)	LC	Urogenital carcinomas	Viral, pollutants, bacteria, genetic	18%	Deming et al. (2018)
Mollusc	Soft shell clam (*Mya arenaria*)	NL	Transmissible cancer	Transmissible cancer	6.1%–95%	Brousseau (1987)
	Mussel (*Mytilus trossulus*)	NL	Transmissible cancer	Transmissible cancer	0%–29%	Mix (1983)
	Cockle (*Cerastoderma edule*)	NL	Transmissible cancer	Transmissible cancer	2%–46%	Poder and Auffret (1986)
	Carpet‐shell clam (*Polititapes aureus*)	NL	Transmissible cancer	Transmissible cancer	42%	Metzger et al. (2016)
	Mussel *(Mytilus chilensis)*	NL	Transmissible cancer	Transmissible cancer	5%–13%	Yonemitsu et al. (2019)
	Mussel *(Mytilus edulis)*	NL	Transmissible cancer	Transmissible cancer	4%	Yonemitsu et al. (2019)
Reptile	Green sea turtles (*Chelonia mydas*)	E	Dermal fibropapillomas	Viral	60%	Jones, Ariel, Burgess, and Read (2016)
	Hawksbill turtle (*Eretmochelys imbricata*)	CE	Dermal fibropapillomas	Viral	Unknown	Poli, Lopez, Mesquita, Saska, and Mascarenhas (2014)
	Loggerhead turtle (*Caretta caretta*)	V	Dermal fibropapillomas	Viral	Unknown	Page et al. (2015)

Note

The conservation status of affected species according to the International Union for Conservation of Nature (IUCN) is shown: CE, Critically Endangered; DD, Data Deficient; E, Endangered; LC, Least Concern; NL, Not Listed; NT, Near Threatened; V, Vulnerable.

## 2 IMMUNE RESPONSES TO INFECTIOUS CANCERS IN WILDLIFE

Infectious cancers can be broadly grouped into two categories: directly transmissible cancers, where the infectious agent is the cancer cell itself (Ostrander, Davis, & Ostrander, 2016), and indirectly transmissible cancers, where the infectious agent is a pathogen such as a virus that induces cancer formation (Ewald & Swain Ewald, 2015). Although there are similarities in terms of host immune responses, the interaction between these types of infectious cancers with the host immune system is multifaceted and in some cases cancer‐specific, as described in detail below. The vertebrate immune system consists of two arms: the innate immune system, which functions to induce systemic inflammation and nonspecific immune responses (Hato & Dagher, 2015), and the adaptive immune system, which exerts specific immune responses against pathogens or tumour‐associated antigens (Cooper & Alder, 2006). Understanding the interaction between infectious cancers and the host immune system is key to developing effective disease management strategies.

### 2.1 Directly transmissible cancers

There are nine known directly transmissible cancers: one in domestic dogs (CTVT; Murgia, Pritchard, Kim, Fassati, & Weiss, 2006), two independently evolved transmissible tumours in Tasmanian devils (*Sarcophilus harrisii*) (devil facial tumour disease [DFTD] and devil facial tumour 2 [DFT2]) (Pearse & Swift, 2006; Pye, Pemberton, et al., 2016) and six lineages of transmissible neoplasia circulating in six species of marine bivalves (Metzger et al., 2016; Yonemitsu et al., 2019). In CTVT and DFTD, immune evasion is at least partially achieved through downregulation of the major histocompatibility complex (MHC) proteins from the tumour cells' surface (Siddle et al., 2013; Yang, Chandler, & Dunne‐Anway, 1987). MHC is a highly polymorphic group of proteins which label infected or cancerous cells for immune destruction by T cells (Wieczorek et al., 2017). Thus, removal of MHC from the cell surface hides the cancer from host immune cells and prevents clearance by the adaptive immune system. It has been demonstrated in both CTVT and DFTD that restoration of MHC to the cell surface can result in specific immune responses against the tumour cells (Hsiao et al., 2008; Tovar et al., 2017). In contrast, DFT2 expresses MHC on the cell surface (Caldwell et al., 2018); however, recent evidence suggests that DFT2 is currently losing its MHC‐I expression from the cell surface (Ong, Lyons, Woods, & Flies, 2019), thereby enhancing its transmissibility potential.

Major histocompatibility complex polymorphism enables immune recognition of many pathogens, ensuring species survival in the face of epidemics (Savage & Zamudio, 2011; Sommer, 2005). Low polymorphism has been linked to reduced species fitness and a lower ability to recognize novel pathogens (Belasen, Bletz, Leite, Toledo, & James, 2019; Maibach & Vigilant, 2019), although this is not always the case (Castro‐Prieto, Wachter, & Sommer, 2010). Low genetic diversity in Tasmanian devil populations, particularly in MHC genes (Cheng et al., 2012; Morris, Austin, & Belov, 2013; Siddle, Kreiss, et al., 2007), may have reduced the ability of the devil's immune system to distinguish self from non‐self‐malignant cells, facilitating the emergence of transmissible tumours (Caldwell et al., 2018). These mechanisms of emergence have been implicated in both DFTD and CTVT (Murchison et al., 2014; Siddle, Sanderson, Sanderson, & Belov, 2007), although the absence of MHC molecules from circulating tumours indicates that the host immune system has exerted pressure on the cancer cells during their evolution, as has been observed in single‐organism cancer (McGranahan et al., 2017). The immune responses seen in DFTD, DFT2 and CTVT hosts are largely tumour‐specific, indicating activation of the adaptive immune system against the cancer cells (Cohen, 1972; Hsiao et al., 2008; Pye, Hamede, et al., 2016; Tovar et al., 2017). It is unclear whether marine bivalves raise any immune response against their transmissible neoplasia, although the lack of an adaptive immune system and MHC in invertebrates suggests that they may be more vulnerable to direct transmission of cancerous cell lines (Gestal et al., 2008; Metzger & Goff, 2016). At least until stronger anticancer defences (resistance) are selected for in these species, individuals could potentially achieve higher fitness by increasing their tolerance to cancer, that is surviving despite the presence of tumours (Thomas et al., 2019). Further studies would be necessary to test this hypothesis and to determine the extent to which the ecological and evolutionary drivers of tumour suppressor gene expression observed in certain vertebrates (i.e. elephants, see Abegglen et al., 2015) are also relevant in invertebrates. Currently, there is no empirical evidence for an exogenous initiator for any clonally transmissible cancers (Metzger et al., 2016; Murchison et al., 2012, 2014; Stammnitz et al., 2018). A promising direction worth to explore in light of the increasing number of transmissible cancers (Metzger & Goff, 2016; Ujvari, Beckmann, et al., 2016; Ujvari Gatenby, & Thomas, 2016bb) is to determine the contribution of the immune system complexity to the emergence of contagious malignant cell lines and whether transmissible tumours have an immune cell originator.

### 2.2 Indirectly transmissible cancers

There are several examples of indirectly transmissible cancers in nature that induce variable host immune responses and are commonly associated with infection by oncogenic pathogens, though additional initiating factors are often implicated in tumorigenesis. In Atlantic bottlenose dolphins (*Tursiops truncatus*) suffering from papillomavirus, there is systemic inflammation and an activated innate immune response, with a partially activated adaptive immune response targeted against the virus rather than the tumour (Bossart et al., 2008; Rehtanz et al., 2010). Similarly, systemic inflammatory immune responses have been observed in green sea turtles (*Chelonia mydas*) suffering from virus‐associated fibropapillomatosis alongside reduced lymphocyte proliferation, which may indicate immune exhaustion and a reduced capacity to raise an adaptive immune response to the tumour or pathogen (Cray, Varella, Bossart, & Lutz, 2001). A similar reduction of T‐cell function has been demonstrated in Tasmanian devils following DFTD infection, suggesting that while the mode of avoiding T‐cell recognition in DFTD is still not fully understood, there are similarities in certain immune evasion mechanisms (Cheng et al., 2019). In California sea lions (*Zalophus californianus*) suffering from Otarine herpes virus‐1 (OtHV‐1)‐associated urogenital carcinoma (King et al., 2002), there is a strong correlation between environmental organochlorine contamination and cancer incidence despite equivalent OtHV‐1 infection rates (Randhawa, Gulland, Ylitalo, DeLong, & Mazet, 2015). A link has also been demonstrated between MHC diversity and cancer risk (Bowen et al., 2005), indicating a genetic component to the disease that mirrors the emergence of directly transmissible tumours (Ujvari et al., 2018). The ceruminous gland tumours affecting the Santa Catalina Island fox (*Urocyon littoralis catalinae*) are associated with ear mite infestations, and a generalized systemic inflammatory environment caused by bite wounds combined with a specific immune response to ear mite infection is thought to encourage tumour formation (Moriarty et al., 2015; Vickers et al., 2015). Similar mechanisms have been suggested in the emergence and transmission of facial tumours in the Tasmanian devil due to their aggressive social interactions (Hamede, McCallum, & Jones, 2013; Stammnitz et al., 2018).

The complex underlying causes of infectious cancers caused by pathogens often result in a systemic and nonspecific immune response that is not protective, causing chronic infection and tumour persistence (Browning, Gulland, Hammond, Colegrove, & Hall, 2015; Moriarty et al., 2015). One common feature that may underpin the emergence of directly and indirectly transmissible cancers is low genetic diversity, as evidenced by Tasmanian devils (Siddle, Kreiss, et al., 2007), Santa Catalina Island foxes (Hofman et al., 2015) and California sea lions (Acevedo‐Whitehouse, Gulland, Greig, & Amos, 2003). However, many wild populations with extremely low genetic diversity thrive without increased cancer incidence (Weber, Stewart, Schienman, & Lehman, 2004), indicating that genetic diversity cannot alone be causative and that more complex interactions may be responsible for carcinogenesis. Although strong associations exist between pathogens and indirectly transmissible tumours, most infected individuals do not develop cancer, indicating that infection alone is not entirely the cause of tumour growth (Rehtanz et al., 2010; Vickers et al., 2015).

Infectious cancers are the result of complex combinations of genetic susceptibility, pathogenic infections, and abiotic and behavioural factors that allow the emergence and transmission of tumour cells or pathogens between individuals (i.e. the “perfect storm,” see Ujvari, Beckmann, et al., 2016; Ujvari Gatenby, & Thomas, 2016aa). Understanding the interplay between these risk factors during the emergence and spread of cancers that are either caused by pathogens or by contagious cancer cell lines will not only help in managing current epidemics but also help to identify and manage emerging epidemics before they become widespread.

## 3 ECOLOGICAL, EPIDEMIOLOGICAL AND EVOLUTIONARY DYNAMICS OF CANCERS IN WILDLIFE

Cancer emergence and progression do not occur in a vacuum, but rather in a complex suite of ecological and evolutionary interactions. In the same way that hosts can compensate for the fitness effects of parasitic infections (i.e. phenotypic plasticity of life‐history traits), cancer is expected to trigger host responses to cope with the immunological and physiological demands of growing tumours. The diverse effects of cancer in host fitness (i.e. vulnerability to predation, susceptibility to coinfection with other pathogens, limited reproductive output, reduced ability to disperse) often result in host responses and adaptive processes early in cancer development. For example, an experimental study demonstrated that drosophila (*D. melanogaster*) with induced colorectal cancer are able to adjust their life‐history traits by reaching the peak of oviposition significantly earlier that healthy ones (Arnal et al., 2017). Furthermore, there is evidence that the social environment of hosts can have a significant impact on cancer progression. Drosophila with induced colorectal cancer had faster tumour growth rates when kept in isolation than did flies in control groups (Dawson et al., 2018). These responses demonstrate the intricate and dynamic relationships between hosts and oncogenic processes and the ability of hosts to trade off fitness costs at different stages of disease. Likewise, host social structure, behaviour and sexual selection have the potential to affect contact rates and hence the transmission of infectious cancers (Vittecoq et al., 2015). Environmental factors driving the emergence of cancers, whether from anthropogenic sources (e.g. carcinogenic pollutants,) or natural sources (e.g. viral oncogenes), suggest a continuum of interactions and selection between host and oncogenic processes. Furthermore, increasing evidence from genomic studies suggests that certain oncogenes are capable of mutating and jumping hosts in species with disparate habitats and environmental attributes (Cortes‐Hinojosa et al., 2019; Literak et al., 2010).

The vast majority of deaths caused by cancer are related to metastases, that is the development of secondary malignancies arising from the primary site of cancer in the host's body. However, metastatic cancer is the endpoint of a much more complex process with several stages, ranging from precancerous lesions to localized establishment and disseminated growths (Vittecoq et al., 2013). In some circumstances, cancerous lesions might never metastasize, either because the hosts die from other causes or due to the development of defence mechanisms such as tolerance and resistance. While tolerance (the ability to reduce disease costs in host fitness) and resistance (the ability to reduce disease burden or eliminate the disease) are mechanisms that have been mostly studied in host–parasite systems, there is now increasing evidence that these defence strategies are also applicable to oncogenic phenomena (Margres et al., 2018; Thomas et al., 2019). This is particularly relevant for transmissible cancers, where malignant cell lines persist beyond the host's life expectancy and selective processes favour the development of coping strategies across generations. For example, Tasmanian devils affected by DFTD have developed tolerance to the tumours, with females being able to maintain body condition at significantly larger tumour volumes than males (Ruiz‐Aravena et al., 2018). Additionally, survival after DFTD infection has increased significantly in long‐term diseased areas (Wells et al., 2017). An example of resistance to cancer occurs in CTVT, which originated in a wild canid between 6,000 and 10,000 years ago and currently affects domestic dogs (Baez‐Ortega et al., 2019; Murgia et al., 2006). Infected dogs are able to develop immune responses, causing tumour regression and recovery (Das & Das, 2000). Although CTVT may have been highly lethal early in its evolutionary history (see also Leathlobhair et al., 2018), it now coexists with its hosts (Strakova & Murchison, 2015). Coexistence between dogs and CTVT might be the result of continuous selective processes between the cancer cell line and hosts over millennia. However, the strong selective pressures of cancer can also operate on extremely short time scales. For example, a small proportion of Tasmanian devils have developed immune responses to DFTD resulting in natural tumour regressions in as little as 8–10 years (4–5 generations) after the cancer epidemic (Pye, Hamede, et al., 2016). The extremely high mortality of DFTD and the subsequent catastrophic population declines resulted in selection in regions of the genome that are associated with immune function and cancer risk (Epstein et al., 2016).

The high mortality caused by DFTD, where almost all individuals die within a year of attaining sexual maturity and their first mating event, would place strong selection pressure on life‐history traits. In response to the cancer epidemic, a significant shift to younger populations and a 16‐fold increase in the proportion of females able to breed in their first year (precocious sexual maturity) has been observed in several diseased sites (Jones et al., 2008; Lachish, McCallum, & Jones, 2009). Likewise, offspring sex ratios are more female‐biased in diseased mothers compared to healthy mothers and litter size per female is significantly larger in populations where DFTD is present (Lachish et al., 2009; Lazenby et al., 2018). The rapid phenotypic and genotypic responses in the Tasmanian devil demonstrate that fast evolutionary processes in response to cancer can occur on ecologically relevant time scales. These processes can occur not just at the host level but also at the tumour level. Despite being a clonal cancer cell line, molecular studies have shown that DFTD is also subject to evolutionary plasticity (Murchison et al., 2012; Pearse et al., 2012; Ujvari, Beckmann, et al., 2016; Ujvari Gatenby et al., 2016aa). More importantly, the evolutionary dynamics in the tumour can affect individuals and populations in different contexts. As the DFTD epidemic unfolded, a sudden local replacement of tumour karyotype (from tetraploid to diploid) resulted in a significant increase of infection rates and population decline (Hamede et al., 2015). Observed differential growth rates between tetraploid and diploid tumours (Hamede, Beeton, Carver, & Jones, 2017) may also select for polymorphism in tumour virulence. This may provide scope for an evolutionary arms race between cancer cells and hosts. At the host level, a broad range of eco‐immunological dynamics such as seasonal dynamics of stress, demographic variation in immune expression profiles, reproductive hormones and immune senescence, as well as genetic and phenotypic variation, may interact with cancer susceptibility and tumour progression. At the tumour level, selection should favour lineages that reach optimal virulence, a trade‐off between transmission rate and disease‐induced mortality (Ebert & Bull, 2003).

**FIGURE 1:**
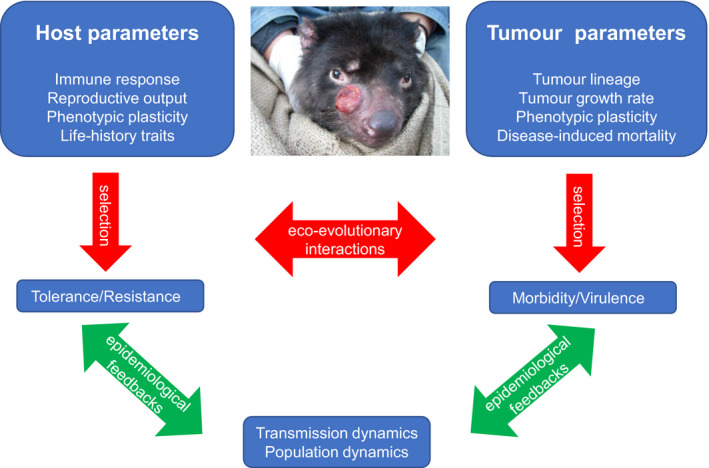
The Tasmanian devil and its transmissible tumour (DFTD), an ideal model system to understand how species adapt and evolve in response to infectious cancers and study the interplay of ecological, evolutionary and epidemiological processes. Blue boxes represent host and tumour parameters under selection through evolutionary interactions (red arrows). Host tolerance and resistance and tumour morbidity and virulence are under selection through ecological and evolutionary interactions. These interactions feed back into epidemiological and population dynamics (green arrows)

The Tasmanian devil–DFTD system provides a unique opportunity to understand the interplay of ecological, evolutionary and epidemiological dynamics in response to cancer (Figure 1). Both tumours and devils have been consistently studied at multiple scales across the species' distributional range since the beginning of the epidemic. The observed selection and eco‐evolutionary dynamics in DFTD should be used as a benchmark for studying and managing DFT2, for which limited information exists (James et al., 2019). More importantly, this knowledge could allow the use of several modelling approaches to predict the evolutionary trajectory of malignant cells as well as evaluating critical epidemiological parameters such as tumour virulence, host susceptibility and tolerance/resistance to infection.

## 4 FOLLOWING THE CANCER FOOTPRINT: FROM SPECIES CONSERVATION TO ECOSYSTEM FUNCTION

Cancer may be of particular concern in the small population paradigm in conservation, where stochastic causes of mortality can present a significant threat. Small populations or threatened species with low genetic diversity might be more susceptible to cancer (Ujvari et al., 2018). Population‐level effects of cancer, such as reduction in population growth rate and cascading effects flowing through community and ecosystem levels, can be difficult to document. Establishing causal links between population decline and oncogenic processes in wildlife is fraught with the difficulties of long‐term investigation and establishing the cause of mortalities in a sufficient proportion of the population. The clearest and best documented case of population decline caused by cancer is the Tasmanian devil–DFTD system (see Box 1).

Box 1 **The birth, spread and impact of transmissible cancers in Tasmanian devils**

**FIGURE 2:**
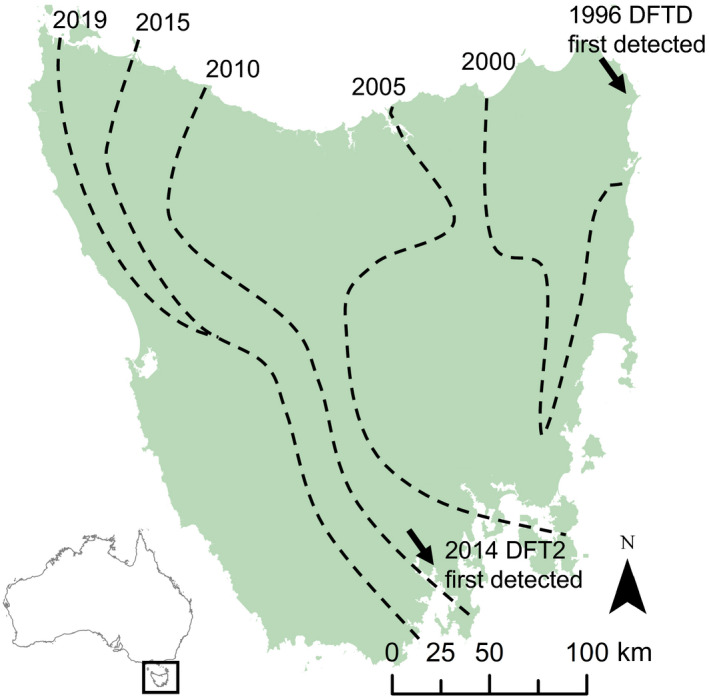
Map of Tasmanians showing the emergence and progression of two transmissible cancers in Tasmanian devils. While DFTD has spread from east to west over the last 25 years (dashed lines), DFT2 is still confined to the geographic area where it was first discovered

First detected in 1996 in northeast Tasmania, DFTD has spread across most of the distributional range of the devil in Tasmania (Figure 2; Hawkins et al., 2006; Lazenby et al., 2018). Clearly visible primary tumours (Loh et al., 2006), high recapture probability in trapping surveys (Lachish, Jones, & McCallum, 2007), and a concerted field monitoring and research effort have enabled clear causal links between DFTD spread, population decline and cascading effects at the ecosystem level to be established (Lazenby et al., 2018; McCallum et al., 2007).

Devil population decline accelerates 3 years after local disease emergence because the infection increases exponentially (McCallum et al., 2009), reaching a 60% decline after 5–6 years and up to a 90% decline in some areas (Lachish et al., 2007; McCallum et al., 2007). The rapid population decline led to the species being listed as *Endangered* at the international (IUCN Red List), national and state levels (Hawkins, McCallum, Mooney, Jones, & Holdsworth, 2009). Strong frequency‐dependent transmission, likely caused by biting during the mating season, also contributed to concerns of extinction as a possible outcome of the epidemic (McCallum et al., 2009). However, to date, no local extinctions have been reported and long‐term diseased populations persist despite high prevalence of tumours (Hamede et al., 2015; Lazenby et al., 2018). The rapid evolutionary response of devils to DFTD (within 4–6 generations) indicates that the adaptive shift is operating on the genetic variation present prior to the DFTD epidemic (Epstein et al., 2016), despite the low genetic diversity (Jones, Paetkau, Geffen, & Moritz, 2004; Siddle, Marzec, Cheng, Jones, & Belov, 2010) resulting from population bottlenecks during the last glacial maximum and during the Holocene (Brüniche‐Olsen, Jones, Austin, Burridge, & Holland, 2014).

In 2014, a second and independently evolved transmissible cancer (DFT2) was discovered at the d'Entrecasteaux peninsula in south‐eastern Tasmania. DFT2 and DFTD coexist in the same population, and a limited number cases of coinfection (both diseases in the same individual) have been reported (Kwon et al., 2018). Both tumours have been reported to be of neuroectodermal origin and most likely evolved from devils in north‐eastern and south‐eastern Tasmania (Stammnitz et al., 2018; Storfer et al., 2018). So far, DFT2 seems to be confined to the peninsula where it was first reported, although monitoring efforts outside of the peninsula have been limited (James et al., 2019). The population response to DFT2 and the epidemiological, ecological and evolutionary interactions between devils and DFTD are currently unknown; however, competition and selective processes are expected to occur at individual and population levels. In that sense, current and future research will be vital to predict epidemiological and evolutionary dynamics in the devil/DFTD/DFT2 study system.

Genetically isolated populations or those affected by other threatening processes can become more susceptible to cancer or mutagenic agents. For example, the critically endangered Santa Catalina Island fox neared extinction from hyperpredation by native eagles facilitated by abundant feral pigs (Roemer, Coonan, Garcelon, Bascompte, & Laughrin, 2001). Santa Catalina island foxes are also highly susceptible to exotic diseases (Crooks, Scott, & Vuren, 2001) and to ceruminous gland tumours, for which chronic inflammation from bacterial and mite infestation may promote tumorigenesis (Vickers et al., 2015). The genetic distinctiveness of this subspecies may predispose it to cancer: it has one of the highest rates of cancer observed in a wild population (Vickers et al., 2015). In further examples, debilitation by oncogenic viruses and tumour‐associated mortality limit population growth in small, isolated island populations of the western barred bandicoot (*Perameles bouganville*) and in a small population of Attwater's subspecies of the prairie chicken (*Tympanuchus cupido attwateri*) (McAloose & Newton, 2009). The prevalence of herpes virus‐associated fibropapillomatosis is increasing in sea turtles, particularly in green sea turtles along the coasts of Florida and the Caribbean and Hawaiian Islands; it is considered to be a contributing factor to the ongoing population decline in these endangered species. Evidence of tumour regression offers a pathway for recovery (Guimarães, Gitirana, Wanderley, Monteiro‐Neto, & Lobo‐Hajdu, 2013; Tagliolatto, Guimarães, Lobo‐Hajdu, & Monteiro‐Neto, 2016) and potentially the evolution of resistance, as is occurring in Tasmanian devils (Epstein et al., 2016; Margres et al., 2018).

Documented evidence of trophic cascades triggered by cancer‐induced population decline is rare and often not known (e.g. Santa Catalina Island fox; Vickers et al., 2015). Again, the Tasmanian devil–DFTD host–pathogen system provides the clearest and best documented case study. The progressive spatial and temporal patterns of devil population decline as DFTD has spread from east to west across the island state provide a rare natural experiment on the influential top‐down role of this apex predator and primary scavenger in structuring Tasmanian ecosystems (Hollings, Jones, Mooney, & McCallum, 2014, 2016). The decline in devil populations has released invasive mesopredators from competition, with cascading effects on the decline in populations of small native mammals (Hollings, Jones, Mooney, & McCallum, 2016). Introduced pest species such feral cats (*Felis catus*) and black rats (*Rattus rattus*) have increased in abundance (Cunningham et al., 2018). While the native mesopredator, the spotted‐tailed quoll (*Dasyurus maculatus*), relax their temporal avoidance of devils when devils are at low density (Cunningham, Scoleri, Johnson, Barmuta, & Jones, 2019), it is possible that competition with the similar‐sized feral cat, which has a higher fecundity (two rather than one litter per year), may counter the competitive release from devils. Cats may be holding the smaller native mesopredator, the eastern quoll (*Dasyurus viverrinus*), in a “predator pit” where cats are at high density. Devil decline may also trigger a disease cascade, as the increased numbers of cats have been associated with a higher seroprevalence of *Toxoplasma gondii* in Tasmanian herbivores (Hollings, Jones, Mooney, & McCallum, 2013). These processes at the species and ecosystem levels highlight the broad‐scale effects of cancer in wildlife and the vital need to document and study its implications for ecosystem functioning.

## 5 APPLICATIONS FOR MANAGEMENT AND CONSERVATION

Studies of cancer in wildlife have been mostly accidental and reactive. There has been a historical lack of consistency in studying tumorigenesis across species and monitoring the broad‐scale effects of cancer in wildlife. With few exceptions, such as Tasmanian devils and DFTD and CTVT in dogs, most studies are limited to a few postmortem examinations. In addition, many oncoviruses are asymptomatic and difficult to diagnose, making the epidemiological efforts needed to detect cancer in wildlife even more challenging. The discovery of eight transmissible cancers in the last 25 years (two in Tasmanian devils and six in marine bivalves) suggests that (a) some species/environments might be particularly susceptible to these type of cancers, (b) there is a relationship between environmental change/disturbance and emergence of transmissible cancers or (c) transmissible cancers may have been more common throughout the evolutionary history of multicellular species, but our ability to detect them has only recently improved due to advances in biotechnology and multidisciplinary efforts in surveying populations. Furthermore, while multicellular organisms have been exposed to oncogenic phenomena throughout their evolutionary history, sudden changes in ecological and environmental conditions may result in a mismatch, promoting the development of cancers in the wild. Experimental studies in model systems can be a valuable tool to further understand mechanisms of selection and fitness costs associated with oncogenesis and tumour progression (i.e. Dawson et al., 2018).

Expanding the range and scope of studies on wildlife cancer is necessary to increase our ability to undertake comparative research at the human–wildlife–domestic interface and their environments. Robust data sets are key to further the development of fields such as comparative oncology and to understanding the prognosis, responses and survival in a broad range of malignancies across tissues, individuals, populations and species. Contrasting the biology and evolutionary ecology of tumours across species will provide a new perspective for understanding patterns of carcinogenesis and help mitigate risks of cancer emergence in the wild. We therefore suggest using a three‐level approach to the study of wildlife cancer that will provide a solid link between fundamental research in cancer biology, eco‐evolutionary processes and management and conservation.

First, increased networking and collaborative studies between disease ecologists and cancer biologists would maximize the capacity to diagnose cancer in the wild (Dujon et al. submitted to this Special Issue). A growing number of wildlife, human and environmental health surveys are undertaken globally at multiple scales, and initiatives such as *One Health* and *Conservation Medicine* are providing a practical link for disciplines that previously worked in isolation. This provides an ideal scenario for coordinating and expanding cancer surveillance at the ecosystem level. In parallel, new technologies and promising biomarkers for neoplasia are becoming valuable cost‐effective diagnostic tools for monitoring cancer prevalence in captive and wild populations (Gourlan, Douay, & Telouk, 2019; Kourou, Exarchos, Exarchos, Karamouzis, & Fotiadis, 2015). The combination of technologies, coordination of surveys and diagnostic capacity offers a new platform to detect cancer in the wild, which will improve our ability to swiftly respond to epizootics and collect critical data.

Second, expertise should be drawn from different disciplines by integrating cancer research in humans, domestic species and wildlife populations to understand cellular, organismal and environmental mechanisms of carcinogenesis and their epidemiological and evolutionary patterns. While evolutionary biology and ecology are disciplines that have been recently integrated into oncology, the relevance of eco‐evolutionary processes for recognizing cancer as an important agent of selection remains to be developed and integrated into wildlife management and conservation. For example, evolutionary principles can be used for disentangling within‐ and between‐host dynamics and trade‐offs in response to cancer. Evaluating the role of infectious cancers as important agents of selection across populations provides a holistic and adaptive framework for understanding the adaptive capabilities of different species in response to oncogenic processes (Russell et al., 2018). The integration of these disciplines will also help to disentangle the biological/environmental mechanisms of cancer emergence and evaluate the diversity and lethality of tumours across taxa.

Finally, the knowledge generated from the cross‐discipline framework should be used to develop adaptive management strategies and general guidelines in response to infectious cancers in wildlife. For example, understanding the long‐term effects of the DFTD epidemic in Tasmanian devils—from its devastating population declines to the resulting functional changes in the genome—is critical for evaluating the extent to which management interventions are required. On the one hand, wildlife managers working with threatened wildlife are often focused on maximizing genetic diversity and reducing inbreeding (i.e. genetic rescue). On the other hand, modern genomic techniques have recently allowed the identification of adaptive genetic variation in response to drastic threatening processes such as disease epidemics or environmental degradations. The notion that rapid evolutionary changes in response to emerging infectious diseases can result in highly adapted genotypes and phenotypes by natural selection has given rise to the hope that populations in dire decline can be rescued through evolution (i.e. evolutionary rescue; Carlson, Cunningham, & Westley, 2014; DiRenzo et al., 2018). The adaptive capacity of Tasmanian devils in response to DFTD at different spatial and temporal scales (Epstein et al., 2016; Hamede, McCallum, & Jones, 2019) suggests that eco‐evolutionary processes need to be thoroughly considered by wildlife managers and that rigorous evaluation of host–tumour interactions should be a priority to improve the conservation prospects of species in the face of epidemics (Hohenlohe et al., 2019).

The holistic vision proposed here is particularly relevant for most species affected by infectious cancers. The eradication of infectious cancers is not usually a plausible outcome; therefore, adaptations to these oncogenic processes are likely to evolve. Given the rapid environmental changes we are facing globally, the carcinogenic contaminants circulating in natural habitats and the increasing overlap among human, domestic and wildlife populations, greater attention should be given to screening for the development of neoplastic diseases across species and environments. In this sense, wildlife cancer can act a sentinel of environmental disturbance and species susceptibility to other threatening processes.

## 6 CONCLUDING REMARKS

Much of the historical understanding of cancer has come from studies of human tumours and experimental research in laboratory mice. Because of this, cancer was until recently perceived as an evolutionary dead end. Studies in wildlife are now providing novel perspectives for understanding eco‐evolutionary processes at the cellular and organismal levels. As we look towards the future, there is a unique opportunity to integrate human, experimental and animal cancer research. The examples provided here highlight that cancer in wildlife is the result of a diverse range of mechanisms, including the emergence of novel cancer cell lines able to result in allograft transmission, an increasing number of virus‐associated oncogenes, environmental change and carcinogenic pollutants. The pervasive nature of cancer in wildlife opens the field for studying the genesis of malignant cells, coping mechanisms at the individual level and transgenerational adaptations at the population level. Understanding how species respond and adapt to oncogenic processes and the trade‐offs of suppressing malignant cell growth at the interface of environmental, ecological and evolutionary burdens should become a priority for oncologists, evolutionary biologists, disease ecologists and wildlife managers.

## CONFLICT OF INTEREST

None declared.

## Data Availability

Data sharing is not applicable to this article as no new data were created or analysed in this study.
